# Lasker~Bloomberg Public Service Award to Michael J. Fox: The challenge of Parkinson’s disease

**DOI:** 10.1073/pnas.2622460123

**Published:** 2026-09-09

**Authors:** Randy Schekman

**Affiliations:** ^a^ HHMI, University of California Berkeley, Berkeley, CA 94720; ^b^ Department of Molecular and Cell Biology, University of California Berkeley, Berkeley, CA 94720

## Abstract

This year’s Lasker~Bloomberg Public Service Award honors the singular achievement of Michael J. Fox, who has devoted much of his life to a philanthropic effort to conquer Parkinson’s Disease (PD), a progressive neurodegenerative disorder that afflicts many millions of patients world-wide. Just a few years after his diagnosis of PD at the tender age of 29, Fox pivoted in part from his enormous success in acting [leading roles in the Back to the Future trilogy (1985–1989) and in the hit National Broadcasting Corporation (NBC) sitcom Family Ties (1982–1989)] to the creation of the Michael J. Fox Foundation (MJFF). Fox’s commitment and integrity have altered the landscape of inquiry into the clinical and basic science of PD and have helped to shape public policies and annually raise private ($400M) and government ($255M) support to find more effective treatments. In the 200 y since PD was first described, and despite recent decades of intense clinical work, only a few therapies have been developed to mitigate some aspects of the movement disorder. These treatments, especially the development of dopamine replacement and agonist drugs and the neurosurgical procedure of deep brain stimulation, have provided decades of limited symptom relief to millions of patients but no enduring therapies that alter the arc of progression of the disease. Now in the 4th decade of his personal battle with PD, Fox and the MJFF stand at the threshold of discoveries and clinical trials that show promise of therapies that go to the root causes of the disease.

## The Public Health and Scientific Challenge

With advances in medicine and public health, our average life span has increased, but at the same time, diseases of aging have become more prevalent. Great strides have been made to extend the lives of those afflicted with cancer and heart disease. Nonetheless, we now face a crisis in health care with the inexorable rise in neurodegenerative diseases for which nothing of enduring value has yet been achieved. Among these diseases, Parkinson’s Disease (PD) is increasing at an alarming rate with consequences that challenge our health care systems. Like a pandemic, PD obeys no geographical boundaries, currently affecting the lives of ~6 million people around the world, rising to perhaps 12 million by 2040. In the US, approximately 40,000 people succumb to this fatal disease every year, and there are currently no treatments that can slow or stop its relentless progression (1). For reasons that may relate to environmental exposure, PD is particularly prevalent in China, where it is estimated that in the next decades over half of the new cases of PD will afflict people in Mainland China (2). As a progressive disorder, PD increasingly robs patients of coordinated movement while also inflicting a number of nonmotor symptoms ranging from cognitive impairment to gastrointestinal issues.

**Figure:**
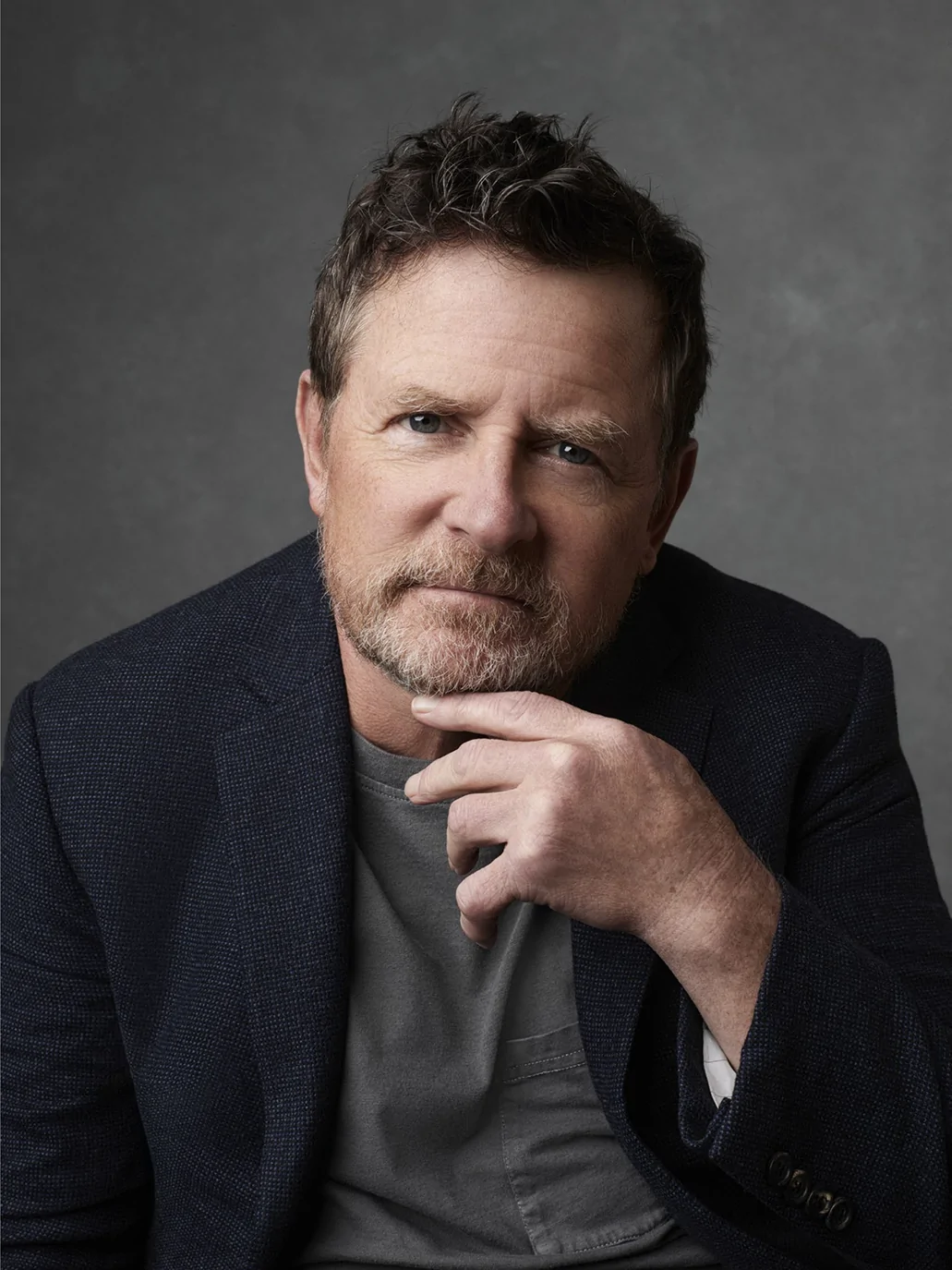
Michael J. Fox. Image credit: Mark Seliger (photographer).

Significant advances in genetic and genomic analysis have uncovered now over 20 genes, mutant alleles of which are found in familial forms of the disease. Mutations in PD genes constitute perhaps 10 to 15% of all forms of the disease. Among the best understood PD genes are LRRK2 a gene which encodes a protein kinase; GBA, which encodes a lysosomal glucocerebrosidase; PINK1 and Parkin which control the disposal of damaged mitochondria; and SNCA, which encodes α-synuclein, an aggregation-prone protein that may account for the spread of brain pathology in both genetic and nongenetic forms of PD. There are likely other genetic contributing factors, with nearly 100 different loci that potentiate the development of the disease (3). The more common idiopathic, or sporadic, forms of the disease may result from exposure to industrial pollutants or environmental factors, some of which are known and could potentially be controlled through legislation (4).

## The PD Brain

PD patients often first present with a movement disorder or a tremor. At that point of diagnosis, which for some patients may be as early as in their 30s, the disease may have already resulted in the loss of half or more of their dopaminergic (DA) neurons. The disease is more like a spectrum disorder where patients progress in different ways and paces, with different sets of symptoms. In a PD patient brain biopsy, the midbrain region shows a loss of the pigment neuromelanin, an oxidation product of the neurotransmitter dopamine. Loss of the pigment reflects the irreversible death of DA neurons in the substantia nigra pars compacta (SNpc) region of the brain.

About a hundred years ago, a German-born American neurologist, Frederic Henry Lewy, discovered in mid-brain sections of patients who died of PD that some neurons had an inclusion now named in his honor. Lewy bodies are a characteristic postmortem indicator of idiopathic forms of PD. Diagnoses of dementia with Lewy bodies and diffuse Lewy body disease are made based on physical/emotional/cognitive symptoms without reference to the actual detection of Lewy bodies. In brain tissue biopsy samples these structures are found as cytoplasmic inclusions within cells, not only in the substantia nigra. As the disease progresses, Lewy bodies may be seen elsewhere in the brain, particularly with those patients who suffer from dementia, which afflicts roughly 30% of patients who have PD. In 2014, the comic Robin Williams took his own life in a desperate reaction to the onset of a rapidly developing form of PD. A biopsy showed Lewy bodies spread through his brain including in the amygdala, likely accounting for his anxiety, paranoia, and intense fear (5).

We now know that the Lewy body includes α-synuclein and may form in response to its aggregation. Although the normal brain function of α-synuclein is not clear, certain mutant forms or higher than normal levels of the wild-type protein promote its aggregation, along with other particles, membranes, and filaments, to form very large, micron-sized inclusions within the cell. What is not yet clear is if the Lewy Body is a by-product of the disease or is the basis of the disease. A similar ambiguity concerns the role of amyloid plaques in the initiation and progression of Alzheimer’s disease. Nonetheless, Lewy bodies have been evaluated in postmortem biopsies of patients who died at various stages in the development of the disease. According to a hypothesis developed by a German clinical scientist, Heiko Braak, there may be a progression of Lewy bodies from the brain stem to regions such as the substantia nigra which house the DA neurons most vulnerable in PD patients. In biopsy specimens of patients who died early in the progression of PD, Braak observed Lewy Bodies in the brain stem and in the olfactory bulb. PD patients often report having experienced a loss of olfactory sensitivity many years in advance of the onset of typical symptoms of PD. Such a condition, known as hyposmia, is now being used to screen individuals at risk for PD to enroll them in clinical studies vital to therapeutic development. In later aspects of the disease, where emotional and cognitive disturbances are more prevalent, these Lewy bodies can be detected in higher regions of the brain (6).

The appearance of and progression of Lewy Bodies correlate with PD providing strong support for the role of α-synuclein in the advance of PD. However, until very recently, it has not been possible to evaluate these structures with noninvasive probes. New work encouraged and supported by the MJFF has led to two major breakthroughs of critical diagnostic value. New PET probes for the detection of α-synuclein deposits in patients with idiopathic forms of PD are now being tested with the goal of following patients as the disease progresses. Other such probes have already proven useful in monitoring the distribution of amyloid plaques in Alzheimer’s patients. Another breakthrough of proven value has been the discovery that cerebrospinal fluid (CSF) samples of patients with an idiopathic form of PD contain a factor that stimulates the aggregation of α-synuclein to form filaments related to the brain forms of aggregated protein in Lewy Bodies. This finding has been turned into a clinical laboratory assay, the α-synuclein seeding assay (SAA) (7). In an ongoing longitudinal study of PD sponsored and funded by MJFF since 2010, the Parkinson’s Precision Medicine Initiative has developed a database of thousands of patients to promote the use of the SAA. The correlation is so strong that the SAA has become the first biological marker to be used to detect the onset and progression of the disease. The SAA has proven to be 87% sensitive with 96% accuracy in detecting PD even before physical symptoms appear. These two markers of α-synuclein aggregation may now be used in clinical trials to assess the efficacy of new drug candidates in arresting progression of the disease (8).

α-synuclein and Lewy bodies are likely to spread within most brain tissues impacted by PD. The basal ganglia, which houses the SNpc, including DA neurons that produce and secrete the neurotransmitter dopamine, and the striatum, which receives input from DA neurons in the SNpc, appear to be the major tissue targets that are responsible for the movement disorder characteristic of PD. A subset of DA neurons in the SNpc appears to be particularly vulnerable. Unlike most brain cells, these DA neurons continuously fire at peak performance to make functional connections to upward of a million nerve cell contacts in the striatum (9). This enormously complicated network of DA nerve connections may be impacted at different points of vulnerability by the diverse genetic and environmental factors that influence disease development.

Two organelles within the nerve cell, the mitochondrion and the lysosome, appear to be particularly vulnerable to the damage inflicted by the spread of aggregates of α-synuclein. Evidence suggests that aggregates of α-synuclein dissipate the electrochemical potential across the mitochondrial inner membrane, reducing the output of ATP (10). This damage is compensated by an upregulation of the glycolytic production of ATP. Nonetheless, in response, cells elicit a pathway of salvage, mitophagy, where defective mitochondria are tagged for traffic to, and destruction in, the lysosome. In healthy cells, two proteins, PINK1 and Parkin, are responsible for the tagging of defective mitochondria (11). Mutations in these genes produce nerve cells that accumulate damaged mitochondria which appear to selectively compromise the viability of DA neurons.

Another genetic factor that may target the lysosome, the LRRK2 gene, appears to have a distinct role in sustaining a signaling connection between a vulnerable DA neuron and a small set of nerve cells in the striatum. Nigral DA neurons communicate with cholinergic interneurons in the striatum through a Hedgehog signaling pathway whose output is detected by a primary sensory cilium on the surface of the interneuron (12). Consequently, activated interneurons secrete growth factors such as glial cell-derived neurotrophic factor (GDNF) that support the survival of DA neurons. Mutations in the LRRK2 gene account for only ~1% of PD patients, but recent studies on how these mutations interfere with DA neuron viability provide valuable clues that may inform how we think about the entire network of influences that impinge on DA neurons. In studies supported by the MJFF in cooperation with the Sergey Brin Family Foundation, teams of investigators at Dundee University in Scotland and at Stanford Medical School have uncovered how PD alleles of the LRRK2 gene interfere with the hedgehog signaling pathway.

The LRRK2 gene encodes a specialized protein kinase that has low enzyme activity in its basal state. LRRK2 mutations in patients, which are genetically dominant alleles, result in an abnormally activated kinase. In this activated state, the kinase phosphorylates a subset of target proteins, Rabs, a large, genetically diverse family of GTP-binding proteins which are known to govern the targeting of membrane vesicles to various destinations in the cell. Phospho-Rabs formed in response to activated LRRK2 interfere with the production of primary cilia on the cholinergic interneurons thus abrogating the Hedgehog signaling pathway and arresting the production and secretion of GDNF. Biopsy material from patients carrying LRRK2 and other phenotypically similar mutations displays a diminished set of primary cilia on the relevant interneurons (13). Treatment of a mouse model of the LRRK2 mutation with a drug that reduces the activity of LRRK2 kinase results in restored growth and function of the primary cilia on relevant cholinergic interneurons in the striatum. Clinical trials are underway to evaluate the possible use of such a drug in restoring DA neuron survival in LRRK2 patients.

## Public and Private Support for PD Research

Breakthroughs in the sciences require resources that can only come from investments provided by governments, venture capital, and philanthropy. In the United States, government funding of basic research started after WWII with a transformative report “Science: The Endless Frontier,” written by Vannevar Bush, the science advisor to Presidents Roosevelt and Truman. He wrote, “Scientific progress on a broad front results from the free play of free intellects, working on subjects of their own choice, in the manner dictated by their curiosity for exploration of the unknown … Freedom of inquiry must be preserved under any plan for government support of science.” Opportunities for education in the United States exploded in the 1960s. The race to space initiated by Eisenhower and Kennedy led to a substantial increase in federal support of science and science education. This investment nourished generations of American and immigrant scholars that made the United States the world-leading powerhouse of 20th century science and technology. The lessons learned from this transformation have spread around the globe with 21st century leading economies, China foremost among them, rising to match and exceed the pace set by the United States.

Now, 80 y after the report by Vannevar Bush, we see the fruits of that investment in a profound growth of science and biomedicine in the United States. Yet, with respect to the diseases of aging, particularly in neurodegenerative disorders, nothing of lasting impact has emerged to stem the progression of Alzheimer’s and Parkinson’s Diseases where estimates suggest an annual death frequency of ~150,000 in the United States alone.

The economic burden of PD exceeded $82B in 2024 with an impact on ~1.2 M patients in the United States. Significant resources have been deployed over recent decades with research funding amounting to ~$650 M/y, nearly 2/3 of which has come from the MJFF and the Parkinson’s Foundation (14). Private industry invests substantial R & D funds amounting to perhaps billions devoted to the development of novel drugs and treatments. More recently, the Sergey Brin Family Foundation has launched another effort, Aligning Science Across Parkinson’s Disease (ASAP), with a 10 y, ~$1B effort focused on the basic science of PD (15). Like Fox, Brin has a personal stake in advancing the cause of PD research. He and his mother share one of the genetically dominant alleles of LRRK2.

To coordinate federal policy on PD, in 2024 Congress passed legislation authorizing the creation of an Advisory Council on Parkinson’s Research Care and Services. As part of the National Plan to End Parkinson’s Project, the Council will advise the HHS Secretary, coordinate government-wide research, advise on improving access to specialized care, and shape the first plan to end Parkinson’s.

Despite this progress and investment, we now face a new challenge in the public and political acceptance of science in the United States. With the current executive leadership of the country, the infrastructure that supports science in this country faces an existential crisis. We are now challenged by an increasing antagonism to science and the scientific method, even to the point of denying the success of the mRNA vaccines in damping the COVID-19 pandemic, and the antiretroviral medications that have reversed the certain death sentence of HIV infection. The Presidential science advisor is not a scientist. The current Secretary of Health and Human Services, who is neither a scientist nor a physician, has embraced the controversial views of vaccine skeptics and HIV/AIDS denialists and has recommended canceling NIH grants supporting infectious disease research (16, 17). He removed all the members of the CDC committee charged with vaccine policy and replaced them with other vaccine skeptics (18). Both he and the NIH Director have dropped support for mRNA vaccines with the demonstrably false accusation that they were not properly tested and are of unproven value.

Academic institutions, where most of the basic science is conducted, are under attack (19). Universities and research institutes have been directed to accept grants with an unsustainably low level of indirect cost, a restriction that has not been imposed on other government support, such as the aerospace and defense industries (20). The Director of the OMB has publicly expressed the view that science can be supported only insofar as it is consistent with current Presidential priorities (21).

We face a sea change in government support that has shocked our colleagues around the world, some of whom have offered a lifeline not unlike the Marshall plan after WWII. Science will continue to advance around the world even as we take a back seat in adjusting to a new reality. Our place may be taken by those countries, such as China, where the government’s investment in basic science continues to grow as our support dwindles. Our children and grandchildren will pay the price.

It is our responsibility as leaders of the scientific community to speak up more forcefully in support of science. Private philanthropy in the United States is crucial but can never replace the essential role of the government in support of biomedical science. We have decades of evidence that investment in basic science pays enormous dividends in improved health outcomes and technological development. The next generation of scientific leadership must now speak out to show that America can still lead the way.

## Data Availability

Previously published data were used for this work (1, 2, 14).
